# Combining temperature-dependent life table data into Insect Life Cycle Model to forecast fall armyworm *Spodoptera frugiperda* (JE Smith) distribution in maize agro-ecological zones in Africa

**DOI:** 10.1371/journal.pone.0299154

**Published:** 2024-05-06

**Authors:** Marian Adan, Henri E. Z. Tonnang, Cojdo E. F. Kassa, Klaus Greve, Christian Borgemeister, Georg Goergen

**Affiliations:** 1 Center for Development Research (ZEF), University of Bonn, Bonn, Germany; 2 International Centre of Insect Physiology and Ecology (*icipe*), Nairobi, Kenya; 3 School of Agricultural, Earth and Environmental Sciences, University of KwaZulu-Natal, Pietermaritzburg, South Africa; 4 International Institute of Tropical Agriculture (IITA), Cotonou, Republic of Benin; ICAR Research Complex for Eastern Region, INDIA

## Abstract

The fall armyworm (FAW), *Spodoptera frugiperda* (JE Smith) (Lepidoptera: Noctuidae), an invasive agricultural pest, has significantly impacted crop yields across Africa. This study investigated the relationship between temperature and FAW life history traits, employing life cycle modeling at temperatures of 20, 25, 28, 30, and 32°C. The development time for eggs, larvae, and pupae varied from 0–3 days, 10–18 days, and 7–16 days, respectively. The optimal temperature range for immature stage survival and female fecundity was identified as 21–25°C, with the intrinsic rate of increase (rm) and gross reproductive rate (GRR) peaking at 25–28°C. Model validation confirmed the accuracy of these findings. The research further projected the Establishment Risk Index (ERI), Activity Index (AI), and Generation Index (GI) for FAW under current and future climates (2050 and 2070) using RCP 2.6 and RCP 8.5 scenarios. Results indicate that RCP 2.6 leads to a reduction in high-risk FAW areas, particularly in central Africa. Conversely, RCP 8.5 suggests an increase in areas conducive to FAW activity. These findings highlight the impact of climate policy on pest dynamics and the importance of incorporating climatic factors into pest management strategies. The study predicts a potential decrease in FAW prevalence in West Africa by 2070 under aggressive climate mitigation, providing a basis for future FAW management approaches.

## Introduction

Crop failure resulting from global climate change poses one of the most significant threats to the agricultural sector [1]. Given agriculture’s high dependence on and vulnerability to climate variations, it becomes imperative for farmers to grasp the scale of climate change [2]. Regions and countries with elevated annual average temperatures, marginal or previously degraded farmlands, and limited developmental resources face a heightened risk of enduring the full brunt of climate change’s consequences on agriculture. The occurrence of insect pests may also be affected by these shifts. Increasing temperatures can have a strong effect on the health, growth, distribution, and population size of insects [3]. Extreme temperatures and precipitation fluctuations, in particular, have a profound impact on the life cycles of insect pests; thus, climatic changes are extremely likely to influence pests [4].

The fall armyworm (FAW), *Spodoptera frugiperda* (JE Smith) (Lepidoptera: Noctuidae), represents a critical example of a new invasive pest species in Africa, whose recent adaptation and spread across the continent, potentially accelerated by climate change, predominantly affects maize crops. Furthermore, its impact extends to other economically vital crops such as rice, sorghum, sugarcane, cotton, and various vegetables. This broad host range not only poses significant challenges for agricultural productivity and food security but also underscores the urgency of our study in developing effective management strategies for this adaptable pest [5]. FAW is a prominent endemic and agricultural pest in the Americas [6]. FAW was first reported in Africa around 2016, marking the beginning of its invasion on the continent [5]. The invasion route is thought to have been through the importation of infested agricultural products. Currently, FAW has spread to most countries in sub-Saharan Africa, posing significant threats to food security and agricultural productivity [5]. The insect’s name, ’fall armyworm,’ derives from the behavior of its larvae, which migrate in a military-like formation, consuming crops and leaving vegetation devastation in their wake [7]. Due to its widespread distribution, FAW is exposed to a wide range of environmental conditions, including variations in temperature, precipitation, and ground composition [8]. FAW’s migratory behavior is influenced by a multitude of factors beyond temperature increases. These include variations in precipitation, which affect habitat and food availability; ground composition impacting breeding sites and plant health; wind currents aiding long-distance movement; availability of suitable host plants driving migration patterns; and biological influences like natural predators, parasitoids, and diseases. All these elements collectively contribute to the spread and impact of FAW [9]. Environmental changes also have an indirect impact on insect pests through their influence on associated natural enemies, interspecies interactions, habitats, and the availability of host plants [10].

As temperatures rise, the development time of insect immature stages decreases, making them less vulnerable to predators and increasing their chances of survival [11,12]. Adults, on the other hand, emerge earlier as the temperature increases, altering flight activity patterns [13]. For the temperate zones, winter mortality decreases when the average temperature rises, affecting population dynamics. At high temperatures, mature insects may experience reduced body size or mass, a phenomenon that can adversely impact the fertility of females [14]. Insects, characterized by their high reproductive rates and short generation durations, exhibit a remarkable capacity to respond swiftly to climate variability and change, surpassing the adaptive abilities of vertebrates [9,15]. Insect species that do not rely on low temperatures to induce diapause and have short life cycles tend to expand their geographic distributions in response to warming. Conversely, those species dependent on colder temperatures may face less favorable habitats, resulting in lower abundance [13,16]. Thus, in general rising temperatures and increasing climate variability, will make pest management considerably more difficult [6].

Understanding *S*. *frugiperda*’s impact on crops under current and future climates is critical for sustainable agricultural productivity and food security. Thus, it is essential to understand the temperature-dependent population development potential in order to comprehend the pest’s population dynamics and implement control tactics specific to agro-ecoregions, especially in light of the predicted changes in global temperatures, which have increased over the last 30 years [16] and are expected to reach 1.1–6.4°C by 2100 [17].

Earlier models have addressed FAW’s geographical distribution based on the relationship between occurrence data and climate-related parameters [18,19], in addition to estimates of its development thresholds Chen et al. [5] recommended research on the impact of varying temperatures on the incidence of FAW. This can be achieved by utilizing a phenology model software such as the Insect Life Cycle Modelling (ILCYM), developedby the International Potato Centre (CIP). Here the phenology of insects is controlled by temperature and is organized according to their life stages [20]. Such information is crucial for risk assessments, forecasts, and management strategies, especially in a changing climate.

Several studies have examined how constant temperatures affect *S*. *frugiperda* development, survival, and reproduction [21–23], though they only predicted the developmental rate using linear and non-linear models and did not simulate its development, death, and fecundity to estimate population increase. To date, non-linear models accounting for varying temperatures in the immature stages of *S*. *frugiperda* have not been employed to forecast pest dispersal and associated risks in Africa. Yet, the development of a complete population model that fully takes Surface air temperature into account would enable more accurate forecasting of the potential for population expansion of FAW as well as seasonal fluctuation across Africa in many different agroecological zones and beyond. It would also assist in the prediction of the future pest status and impact of *S*. *frugiperda* under climate change. Therefore, our study aimed to estimate (i) the development rate of S. frugiperda under various constant temperatures, (ii) the duration required to complete each developmental stage, (iii) the mortality rate at different stages, and (iv) the egg-laying duration, which is the period from when the female starts laying eggs until she ceases (oviposition period), as part of the overall development cycle from egg to adult. The resulting phenology model will help identify regions where FAW permanent establishment is likely, estimate the number of generations per year, and provide insights into the potential damage under different climate scenarios for the present, 2050, and 2070 in Africa.

## Materials and methods

### Study site

**Fig 1:**
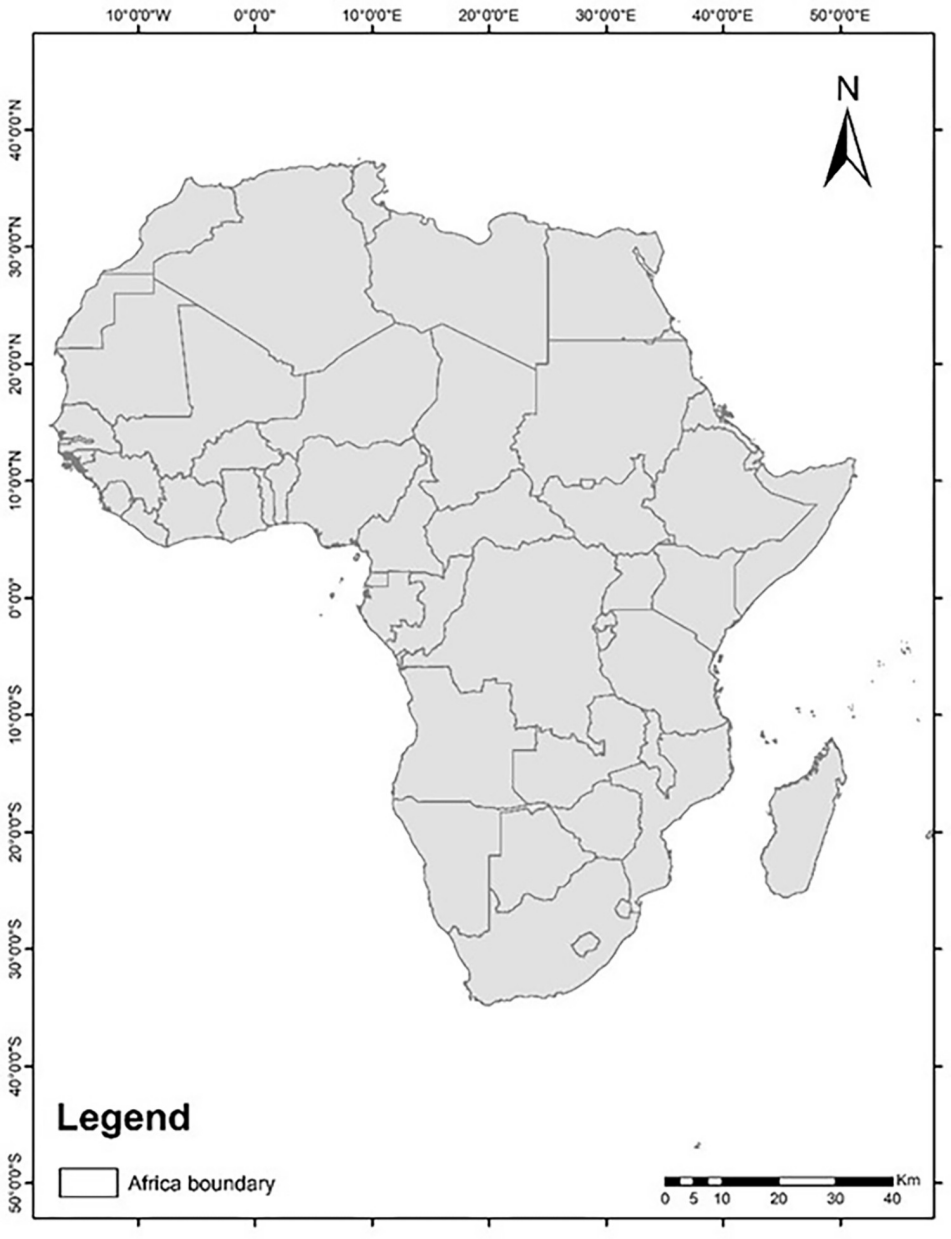
Study areas map showing the African continent boundaries.

Africa is a vast continent that covers approximately 30 million square kilometers and comprises 54 countries (Fig 1). It has a diverse landscape that includes deserts, forests, grasslands, and mountains. The continent’s climate varies from region to region, with some areas experiencing high temperatures and low rainfall, while others have more moderate temperatures and higher levels of precipitation. According to a report by the Intergovernmental Panel on Climate Change (IPCC) [24], Africa is expected to experience an increase in temperature of between 1.5°C and 3°C by the end of the century [17]. The continent has a diverse range of ecosystems, including savannahs, rainforests, and deserts, which are home to a wide variety of plant and animal species [25]. In terms of rainfall, Africa experiences a range of precipitation levels depending on the region. Some areas, such as the Sahara Desert, receive very little rainfall, while others, such as the Congo Basin, receive high levels of rainfall throughout the year. Approximately 40% of the African population lives in areas with high water stress, making water scarcity a significant issue on the continent [25].

### The methodology framework of the study

**Fig 2:**
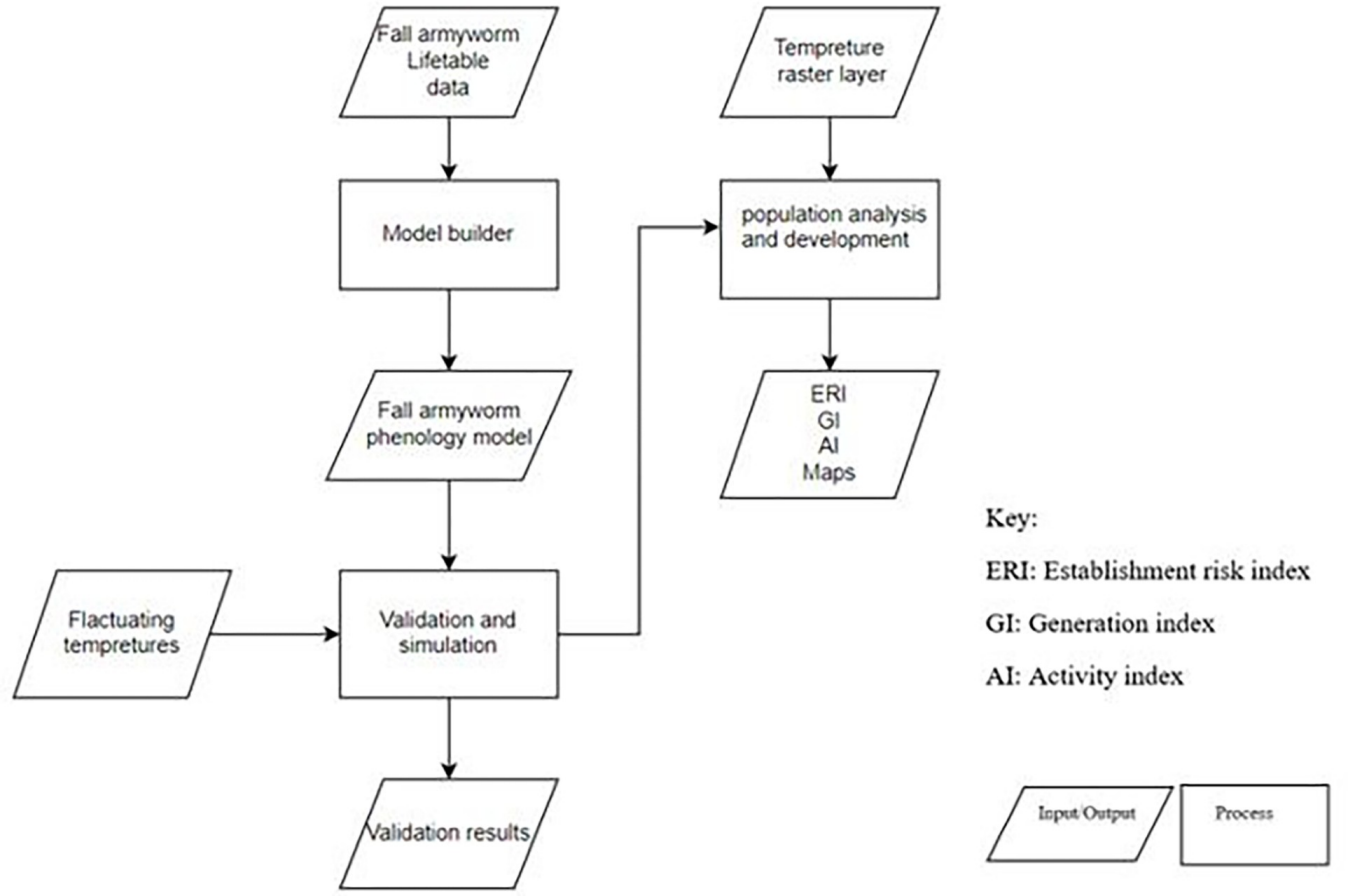
Flowchart of the hierarchical methodology for the analysis of fall armyworm phenology.

The flow chart (Fig 2) describes a methodology for assessing the risk and population dynamics of the fall armyworm based on temperature and lifecycle data. While acknowledging the importance of precipitation as an environmental factor, the study concentrates solely on the impact of temperature variations on FAW. The exclusion of precipitation from our current modeling is a reflection of our focused research objectives and does not diminish the recognized importance of precipitation in the broader context of FAW ecology. The process begins with the collection of life table data on the fall armyworm, which is used in a model builder to create a phenology model for this pest. This model predicts the development stages of the fall armyworm based on temperature data, which is provided by a temperature raster layer.

### Experimental procedures of data collection

#### FAW stock colony

The initial fall armyworm colony of the mass production unit at the Benin station of the International Institute of Tropical Agriculture (IITA) was established in 2018 with caterpillars and egg masses collected on maize in southern Benin and was regularly rejuvenated with fresh field material. This colony followed a standardized rearing protocol widely recognized in entomological research, ensuring consistency and replicability of the experimental conditions. Mass rearing of the larvae was carried out in large plastic containers (18 cm diameter x 10 cm height) lined on the inner side with tissue paper to maintain humidity. The larvae were reared with a 50:50 mixture of sprouted maize grains (Benin, variety EVDT) and germinated cowpea beans (Benin, variety Pkodji) until pupation. Pupae were transferred in new containers for eclosion. Upon emergence, adults were fed with cotton balls soaked in 10% (V/V) honey/water solution and allowed to mate. The containers were covered with gauze on which oviposition took place. Colonies were maintained under natural photoperiod conditions and at laboratory ambient temperatures of 26°C and 83% RH. Crucial growth stages were monitored with a micrometer under a stereoscopic binocular for accurate measurement.

#### Larval development, age-specific fecundity, and adult longevity

All life table data were obtained using climate chambers (Intelligent Artificial Climate Chamber, RTOP-500Y, Zhejiang, China) that were regulated at five constant temperatures (20, 25, 28, 30 and 32°C ±1°C) and 70±5% RH with a photoperiod of 12L:12D. The set temperatures and RH were monitored throughout the experiment with HOBO loggers at 30 min intervals. For each temperature level, freshly laid FAW egg masses were incubated in a plastic container of 130 cm^3^ vol. lined with paper tissue until the eggs hatched. A total of 100 neonate caterpillars were then cautiously transferred with a fine camel hairbrush to individual plastic cups of 30 cm^3^ vol. The larvae were fed with sprouted maize that was renewed every second or third day until larvae pupated. The development of the caterpillars was checked by daily measurement of the cephalic capsule and the presence of exuviae under a stereoscopic binocular. Pupae were sexed and maintained in moistened tissue paper to develop into adults. Newly hatched moths were paired to ensure mating and maintained in a plastic container of 130 cm^3^ vol. until their death. For each temperature level, 30 males and females were subjected to testing. Adult moths were provided cotton balls previously immersed in a 10% (V/V) honey/water solution and females were allowed to oviposit on tissue paper that lined the plastic container. Deposited eggs were counted at daily intervals and longevity monitored until the death of the last adult.

### Statistical analysis and phenology modeling

The open access ILCYM software (version 3.0 - http://www.cipotato.org),developedby CIP [20], was utilized to create a temperature-dependent phenology model of *S*. *frugiperda*. We used ILCYM’s modules for the phenology model by utilizing the ‘model builder’, ‘validation and simulation ‘and ‘population analysis and mapping tools. The software’s model builder has multiple empirical linear and nonlinear models which are utilized to analyze impacts of temperature on insect development. The phenology model is validated using life table data obtained at varying temperatures in the validation and simulation module, and the impact of climate change on the distribution and abundance of the pest is predicted using the population analysis and mapping module. The validation and simulation module takes the validated phenology model as input and processes it through deterministic and stochastic simulations to estimate the life table parameters that determine the pest population’s growth rate. Here we utilized the model builder, validation, and simulation modules to create and test the phenology and to simulate the life table parameters. After fitting a total of 59 non-linear models for each development parameter. Non-linear models are particularly suited for ecological data as they can more accurately capture complex biological phenomena where the relationship between variables is not strictly proportional. These models are essential in our context, where temperature effects on pest development exhibit non-linear characteristics, such as variable growth rates and survival probabilities at different temperature ranges. The most appropriate model for each parameter was selected based on the coefficient of determination (R^2^) and Akaike’s Information Criterion (AIC) [20]. Each fitted parameter in ILCYM was subjected to the Least Square Design (LSD) test at a significance level of 0.05 to establish probability cutoffs. The effects of temperature on the immature growth period, growth rate, mortality rate, male and female longevity, senescence, and oviposition on *S*. *frugiperda* are summarized in Table 1. To ensure the greatest consistency of the phenology model, all larval developmental stages (instars 1 to 6) were combined to create the larva stage, while the pre-pupa and pupa stages were merged into one pupa stage.

**Table 1:** A complete set of mathematical model equations utilized in ILYCM software to develop S. *frugiperda* phenology for each life stage.

Life history traits	Model Name	Equation	Life Stage
Development time	logit	f(x) = 1/ (1 + exp (− (ai + b ln x)))	Egg, Pupa, Female, and Male
Development rate	Logan 5	r(T) = alph. (1/(1+k.exp(-b.x))—exp(-(Tmax-x)/Dt))	Egg
	Hilber & logan 2	r(T) = trid. ((xē)/(xē+D)—exp(-(Tmax-x)/Dt))	Larva
	Logan 1	r(T) = Y(exp(p·T)-exp(p·Tmax-(Tmax-T)/v))	Pupa
Mortality rate	Wang2	y ~ 1–1/ (exp ((1 + exp (-(x—Tl)/B)) * (1 + exp (- (Th—x)/B)) * H))	Egg
	Wang1	m(T) = 1–1/ (exp ((1+exp (-(x-Topt)/B)). (1+exp(-(Topt-x)/B)).H))	Larva
	Gaussian with log	m(T) = y0+a·exp(-0.5(log(abs(x/x0))/b) ^2^)	Pupa
Senescence	Hilbert & Logan 3	y ~ trid * (((x—Tmin) ^2)/ ((x—Tmin) ^2 + D)—exp (- (Tmax—(x—Tmin))/Dt)) + Smin	Female
	Exponential Simple	y ~ b1 * exp (b2 * x)	Male
Relative oviposition	Exponential modified 1	y ~ (1—exp (-(a * x + b * x^2 + c * x^3)))	Female
Total oviposition	Simple gaussian	y ~ y0 + a * exp (-0.5 * ((x—x0)/b) ^2)	Female

#### Development time

For FAW’s developmental periods and adult longevity (in days) at each temperature level the cumulative frequency distributions were fitted to a cumulative distribution function against normalized development times. The logit function was best for egg, larva, pupa, female, and male, and (Table 1) shows the formulae and the parameters of the function utilized [12].

#### Development rate

The development rate of *S*. *frugiperda* was determined by taking the inverse of the median development time and using that value as the basis for the calculation. Stinner et al. [26] employed non-linear models to predict the link between temperature and development rate for various embryonic stages when temperatures were extremely high. For each and every stage of development, the non-linear model [27] was chosen. When compared to the other models in the ILCYM software, the model with the highest R^2^ and the lowest AIC values was selected as the best-fit model. This allowed us to determine which model was the most accurate representation of the data. The Logan-5, Hilber & logan 2, and Logan-6 were found to be the models that best fitted the egg larva and pupa stages, respectively (for the mathematical formula see Table 1).

#### Mortality rate

The mortality rate of *S*. *frugiperda* immature stages at each constant temperature was then used to fit 49 non-linear models. Wang1 and Gaussian with log functions for the egg, larva and pupa stages, respectively [28] proved to be the models that best described how the mortalities of FAW’s immature stages were dependent on the different temperatures (for the mathematical equation see Table 1).

#### Female fecundity and adult senescence

The cumulative fertility of FAW females was plotted against age (for the mathematical formula see Table 1), and was used to fit the cumulative proportion of eggs produced at each age to Exponential modified 1. We also used Simple Gaussianto characterize temperature-dependent fecundity, which we did by fitting the number of eggs laid by each female at each constant temperature. Additionally, we calculated adult senescence (female and male) for each constant temperature by reversing the adult longevity and fitting the data to an exponential simple function (Table 1).

#### Lifetable parameter

The FAW phenology model for development, mortality, female fecundity, and adult senescence were built based on temperature data. Then, the stochastic simulation’ module in ILCYM was used to estimate the following life table parameters: (1) the gross reproductive rate (GRR), (2) the net reproductive rate (Ro), (3) the intrinsic rate of natural increase (r_m_), (4) the mean generation time (Tc), (5) the doubling time (Dt), and (6) the finite rate of increase (λ) at the tested temperatures [20]. Five sets of simulations were run at each temperature, beginning with 100 individuals in the egg stage followed by 30 males and females for the adult stage.

### Model validation

The phenology model was validated by utilizing the fluctuating temperature data that was obtained by recording the daily maximum and minimum temperatures of the experimental site where the *S*. *frugiperda* life table was created under ambient conditions. The Daily varying temperatures data for the period from 01/01/2019 to 31/12/2021 were gathered from the records of the IITA station in Cotonou, Republic of Benin (latitude 6.417127 and longitude 2.329207). The validation model was executed with the data obtained under fluctuating temperature experiments. Next, the phenology model and fluctuating temperatures were used to simulate *S*. *frugiperda* growth and life table characteristics.

To recreate these conditions, we derived a life table from the created phenology model, which included 100 individuals in the egg stage. The results of this procedure (phenology model simulation values) were compared with life table data collected under varying environmental conditions. The constructed phenology model was validated by comparing the differences between observed and simulated values. The constructed phenology model’s validity was confirmed by the consistency between observed and simulated values, suggesting it might be utilized in future research to predict the spread and abundance of the pest in a warming climate.

### Mapping of the potential distribution of *S*. *frugiperda* under current climate scenarios

The "population distribution and risk mapping" module of ILCYM which is integrated with a simple geographic information system (GIS), was utilized to visualize the possible risk posed by *S*. *frugiperda* [20,29]. Using the estimated life table parameters, the establishment risk index (ERI), generation index (GI), and activity index (AI) were derived to indicate the risk of FAW at each location. These equations were utilized to estimate the risk indices.


$$ERI=\frac{\sum_{1}^{i=365}I_{i}}{I_{I}}*net-reproduction$$ (1)

Where, Ii is the interval of day i (with i = 1, 2, 3,…, 365) and the total number of intervals, I_I_, is 365.


$$GI=\frac{\sum_{x=0}^{365}\frac{365}{Tx}}{365}$$ (2)

Where, Tx is the predicted generation length in days at Julian day x (x = 1, 2…,365)

$$AI=\log_{10}\prod_{x=1}^{365}\lambda x$$ (3)

Where, λx is the finite rate of increase at Julian day x (x = 1, 2…,365)

Climate baseline information was interpolated utilizing the worldclim database (http://www.worldclim.org) and subsequently employed in the simulations. To represent the current scenario spanning from 1950 to 2000, as well as the future climate projections for 2050 and 2070 under two Representative Concentration Pathway (RCP) 2.5 (reflecting more ambitious climate mitigation efforts) and 8.5 (indicating more pessimistic climate change scenarios), long-term temperature records from various locations worldwide were used. These records were instrumental in deriving both minimum and maximum temperature values for each month, forming the foundational dataset [30]. The study utilized the output from the ACCESS1-0 GCM model to represent both the current scenario and future climate projections for 2050 and 2070 under RCP 2.6 and RCP 8.5. The choice of ACCESS1-0 was predicated on its widespread application in climate research and its suitability for the spatial and temporal scales of our study.

## Results

### Development time

The duration of *S*. *frugiperda* development was significantly influenced by temperatures (Table 2). The maturity time decreased from low to high temperatures until 32°C, after which development became non-linear. All phases of the development proceeded faster at 32°C than at 20°C. A logit distribution model was used to characterize the variation in maturation durations throughout all phases of the organism’s life cycle (Table 2). Coefficient of determination (R^2^) values varied from 0.97 at the adult male stage with a slope of 8.63 to 1.0; at the egg stage this quantity varied with a slope of 139.46. Across the board, R^2^ and slope values dropped as senescence ages increased (this was true for both sexes).

**Table 2:** Mean development times (days) of immature stages and senescence times (days) of adult life stages of *S*. *frugiperda* at different constant temperatures in the lab. R^2^—Coefficient of determination and AIC -Akaike Information Criterion.

Intercept (a)	Temperature(˚C)	Egg		Larva		Pupa		Female		Male	
		Predicted	Observed	Predicted	Observed	Predicted	Observed	Predicted	Observed	Predicted	Observed
	20	2.449	3	17.891	18	15.720	16	10.262	11	8.402	9
	25	1.414	2	11.446	12	10.696	11	9.105	10	9.294	10
	28	0.805	0	9.641	10	7.680	8	6.961	8	5.737	7
	30	0.805	0	9.255	10	6.526	7	5.789	6	4.895	5.5
	32	0.805	0	9.531	10	6.237	7	4.444	5	4.112	5
Statistics											
Slope (b)		139.46		169.36		21.70		10.63		8.63	
R^2^		1.0		0.98		0.99		0.99		0.97	
AIC		12		169.36		21.70		190.93		250.85	

### Development rate

**Fig 3:**
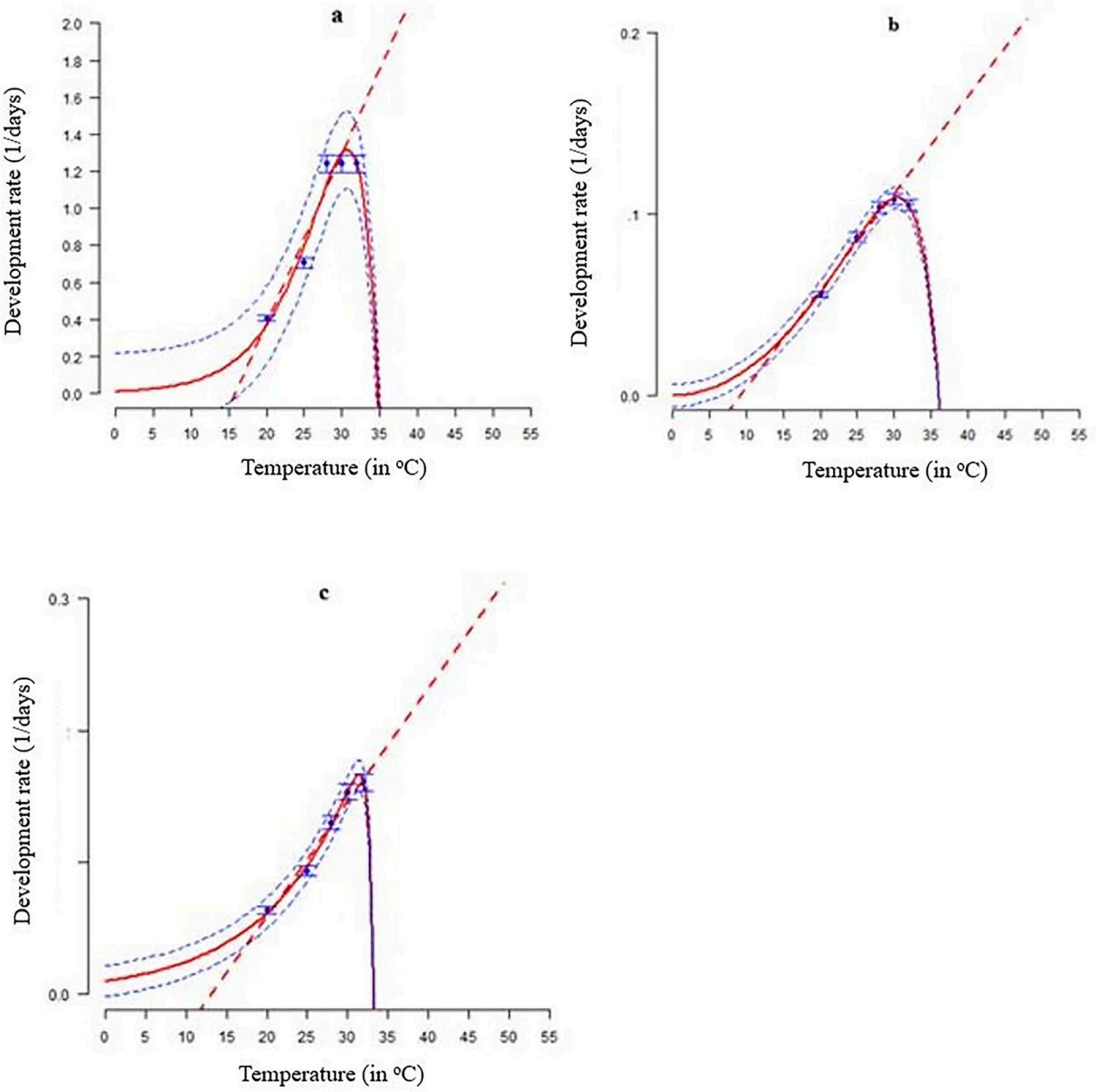
*Spodoptera frugiperda* (a) egg, (b) larva, and (c) pupa development rates as a function of temperature, respectively fitted by Logan-5, Hilber & logan 2, and Logan 1, respectively, at each developmental stage. The standard deviation is depicted by the blue bars, while the blue points indicate the experimental data. The red broken lines represent the fitted linear models, whereas the solid lines represent the fitted non-linear models. Upper and lower 95% confidence intervals are depicted by the broken blue lines above and below, respectively.

Temperature greatly affected *S*. *frugiperda* immature stage development (Table 3 and Fig 3). The linear model fitted all immature phases’ development effectively (R^2^ between 0.95 and 0.99). Logan-5, Hilber & logan 2, and Logan-6 were best for egg, larval, and pupal stages, respectively, among the 59 non-linear models fitted to temperature-dependent development rate (Table 3 and Fig 3A–3C). The best-fit model was the larva stage with a R^2^of0.99(Table 3 and Fig 3B).

**Table 3:** Estimated parameters of the Logan-5, Hilber & logan 2, and Logan 1 models that were used to estimate how temperature affects the rate of development of immature *S*. *frugiperda* that were raised at different constant temperatures. R^2^—Coefficient of determination, AIC—Akaike Information Criterion and df–degree of freedom.

Stage	Function	Model parameters		df	P-value	R^2^	AIC
Egg	Logan5	alph	9.72767 ± 0.1441	4	<0.0001	0.952	5.98
		k	939.4538 ± 0.00484				
		b	0.18416 ± 0.01101				
		Tmax	37.56302 ± 1.20556				
		Dt	2.9495 ± 1.34378				
Larva	Hilber & logan 2	trid	22598.24822 ± 0.02562	3	<0.0001	0.985	-30.68
		D	157157056.08524 ± 1e-05				
		Tmax	67.76324 ± 2.89286				
		Dt	2.71287 ± 0.21605				
Pupa	Logan 1	Y	0.00976 ± 1e-05	3	<0.0001	0.985	-24.198
		Tmax	33.23127 ± 1e-05				
		p	0.09202 ± 1e-05				
		v	0.58139 ± 1e-05				

K—Degree-day (DD) requirements; Y, p and v: Constants values; “P” refers to the number of model parameters while “n” and “m” are constants values; Tmax—The maximum lethal temperature; Dt–the thermal time required for development to be completed.

### Mortality rate

**Fig 4:**
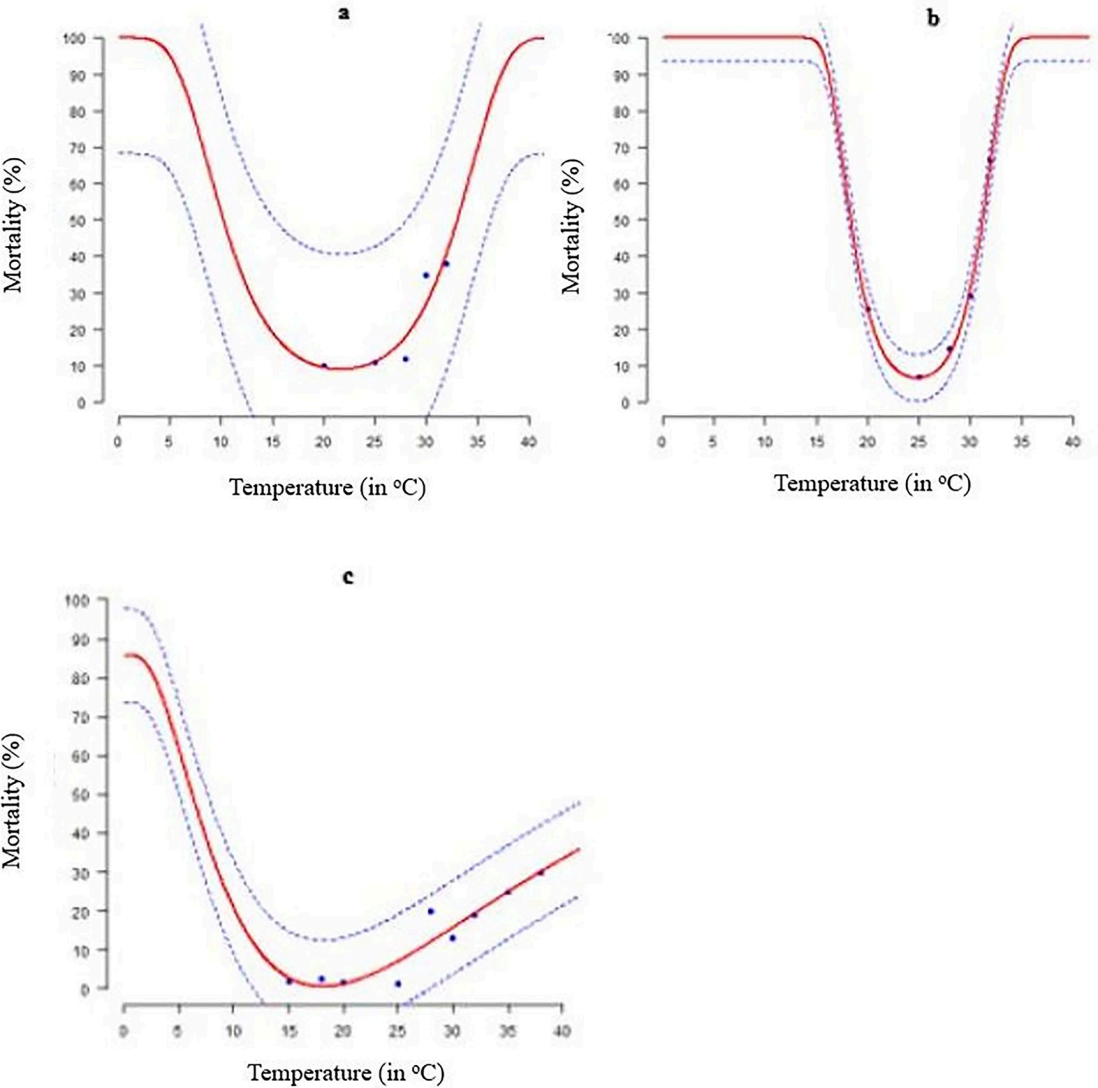
Temperature-dependent death rates of *S*. *frugiperda* immature stages fitted to Wang1, Wang 2 and Gaussian with log models: (a) egg; (b) larva; (c) pupa. The experimental data is blue. Solid red lines represent non-linear models and broken blue lines the upper and lower 95% confidence intervals.

Temperature had a major impact on the mortality rate of the different *S*. *frugiperda* life stages (Table 4 and Fig 4). At 32°C and 20°C, death rates were found to be the highest and lowest across the board, respectively (Fig 4). Wang1 and Wang 2functions for egg and larval stages accurately predicted the temperature dependence of mortality in FAW immature stages (Table 4 and Fig 4). Using this function, we calculated the optimal temperature (Topt), i.e., the lowest mortality rate for the egg (at 21.64˚C), the larva (at 24.79˚C), and the pupa (at 18.09˚C) (Table 4 and Fig 4).

**Table 4:** Estimated parameters of the Wang 2, Wang 1, and Gaussian with log models that were used to estimate how temperature affects the mortality rate of immature *S*. *frugiperda* raised at different constant temperatures. R^2^—Coefficient of determination, AIC—Akaike Information Criterion and df–degree of freedom.

Stage	Function	Model parameters	df	P-value	R^2^	AIC
Egg	Wang 2	Tl 21.64409 ± 6.44452	3	0.46	0.86	-6.47
		Th 21.64362 ± 6.44489				
		B 3.43643 ± 2.00857				
		H 0.02341 ± 0.01904				
Larva	Wang 1	Topt 24.7892 ± 0.08442	2	< 0.0001	0.99	-24.49
		B 1.75891 ± 0.06445				
		H 0.01712 ± 0.00184				
Pupa	Gaussian with log	y0 0.8559± 2.16577	3	< 0.0001	0.89	-24.95
		a -0.8503± 2.15051				
		x0 18.0909± 2.26521				
		b 0.8041± 1.33022				

Where, (Tl) is the minimum lethal temperature, (Th) is the maximum lethal temperature, Topt is the optimal temperature for survival, while B and H are constant values of model parameters.

### Female fertility and adult senescence

**Fig 5:**
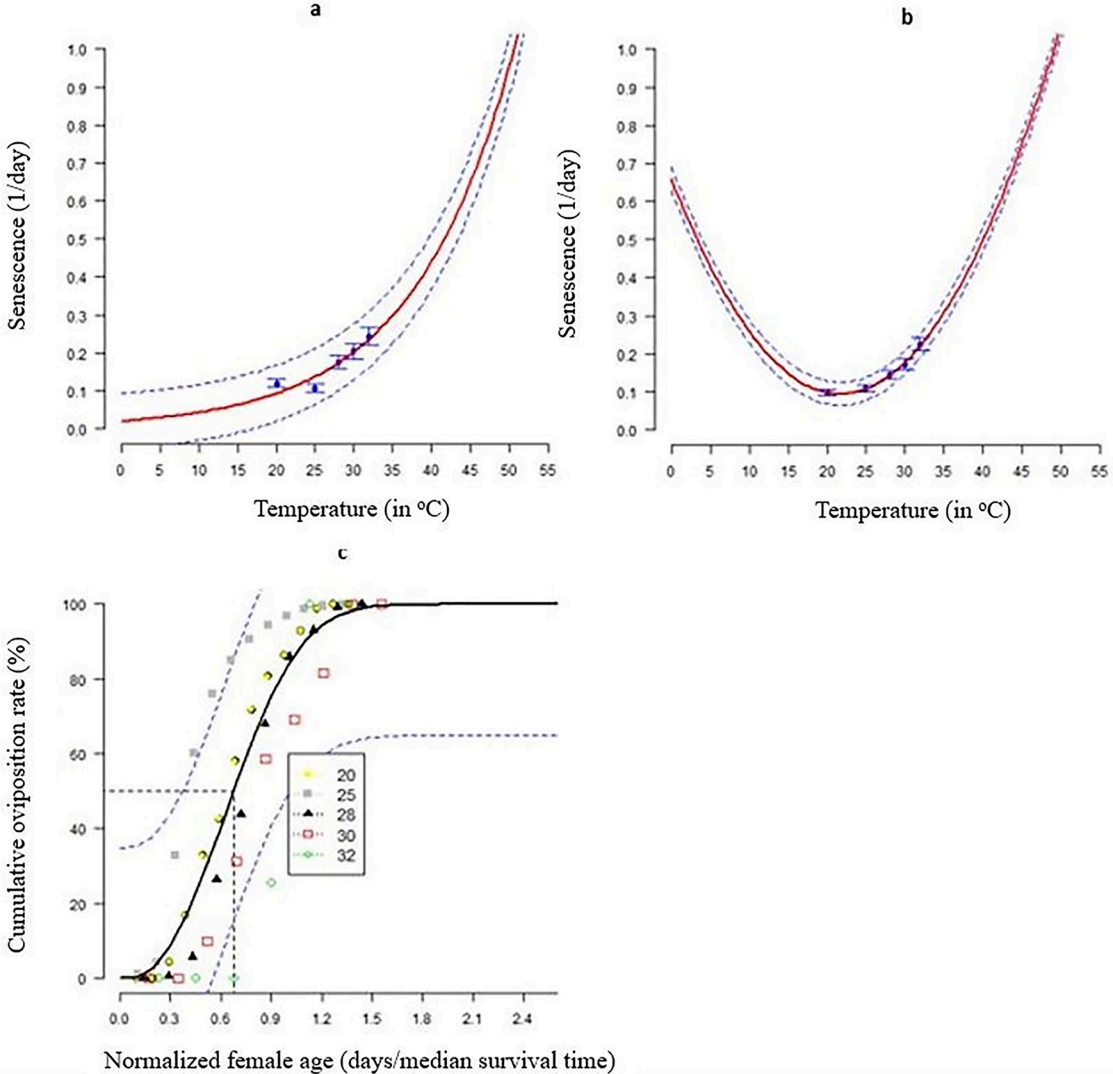
Model fitting to determine the relationship between temperature and *S*. *frugiperda* female fecundity, and adult senescence: (a) female senescence rates fitted to Exponential simple function; (b) male senescence rates fitted to Hilber &Logan 3 function; c) cumulative fecundity fitted to Simple gaussian; and (d) mean fecundity per female fitted to Taylor function 1. The blue points represent the experimental data with bars representing the standard deviation. The solid red lines represent non-linear models. The broken blue lines above and below represent the upper and lower 95% confidence intervals.

Exponential modified function1(R^2^ = 0.82, AIC = -29.29) accurately predicted *S*. *frugiperda* females’ cumulative fecundity based on age (Table 5 and Fig 5C). Temperature greatly affected FAW fecundity (Table 6), with most eggs per female (851.58) yielded at25°C (Table 6 and Fig 5D). Simple gaussian function achieved the best fit temperature-dependent fecundity model (R^2^ = 0.99; AIC = 30.87). The function suggested that *S*. *frugiperda* oviposition was best around 22–23°C (Fig 5D). Temperature affected FAW senescence in both sexes (Table 7). Exponential simple function and Hilber &Logan 3well-described temperature-dependent senescence in both females and males, respectively (Fig 5A and 5B).

**Table 5:** Estimated parameters of the relative oviposition of *S*. *frugiperda*. R^2^—Coefficient of determination, AIC—Akaike Information Criterion, and df–degree of freedom.

Analysis	Formular	Model parameters		df	P-value	R^2^	AIC
Relative oviposition	O(E) = 1-exp(-aE-bE^2-cE^3))	a	-0.11825	2	< 0.0001	0.82	-29.29
		b	1.17905				
		c	0.76887				

Where, parameter ’a’ describes the rate of increase in oviposition as temperature increases, ’b’ describes the temperature at which maximum oviposition occurs, and ’c’ describes the rate of decrease in oviposition as temperature increases above the maximum.

**Table 6:** Estimated parameters of the total oviposition of *S*. *frugiperda*. R^2^—Coefficient of determination, AIC—Akaike Information Criterion and df–degree of freedom.

Analysis	Formular	Fecundity (temp)	Model parameters	df	P-value	R^2^	AIC
Total oviposition	f(T) = y0+a·exp(-0.5((x-x0)/b) ^2^)	20: 776.3889	y0–88.5085	3	0.016	0.99	35.8692
		25: 851.5870	a 1005.384				
		28: 581.5806	b 5.4884				
		30: 353.7500	x0 23.0144				
		32: 176.4444					

Where, parameter ’y0’ describes the minimum oviposition rate, ’a’ describes the rate of increase in oviposition as temperature increases, ’b’ describes the temperature at which maximum oviposition occurs and ’x0’ describes the temperature below which oviposition does not occur.

**Table 7:** Estimated parameters of Hilber & Logan3 and Exponential simple fitted to determine the relationship between temperature and adult senescence *S*. *frugiperda* reared at different constant temperatures. R^2^—Coefficient of determination, AIC—Akaike Information Criterion, and df–degree of freedom.

Life stage	Function	Model parameters		df	P-value	R^2^	AIC
Female	Hilber & logan 3	trid	992834.7	5	< 0.0001	0.95	-11.956
		Tmax	38.567				
		Tmin	21.6748				
		D	8.27E+08				
		Dt	0.0172				
		Smin	0.0949				
Male	Exponential simple	b1	0.02	1	0.01	0.88	-20.04
		b2	0.0773				

Where, Tmin: the minimum temperature required for development to occur; Tmax: maximum temperature above which development is not possible; Trid: the thermal time required to initiate development; Dt: Doubling time; D: the proportion of the development time that has already occurred; b1: the thermal time constant for development; and b2: the baseline temperature for development.

### Life table parameters

**Fig 6:**
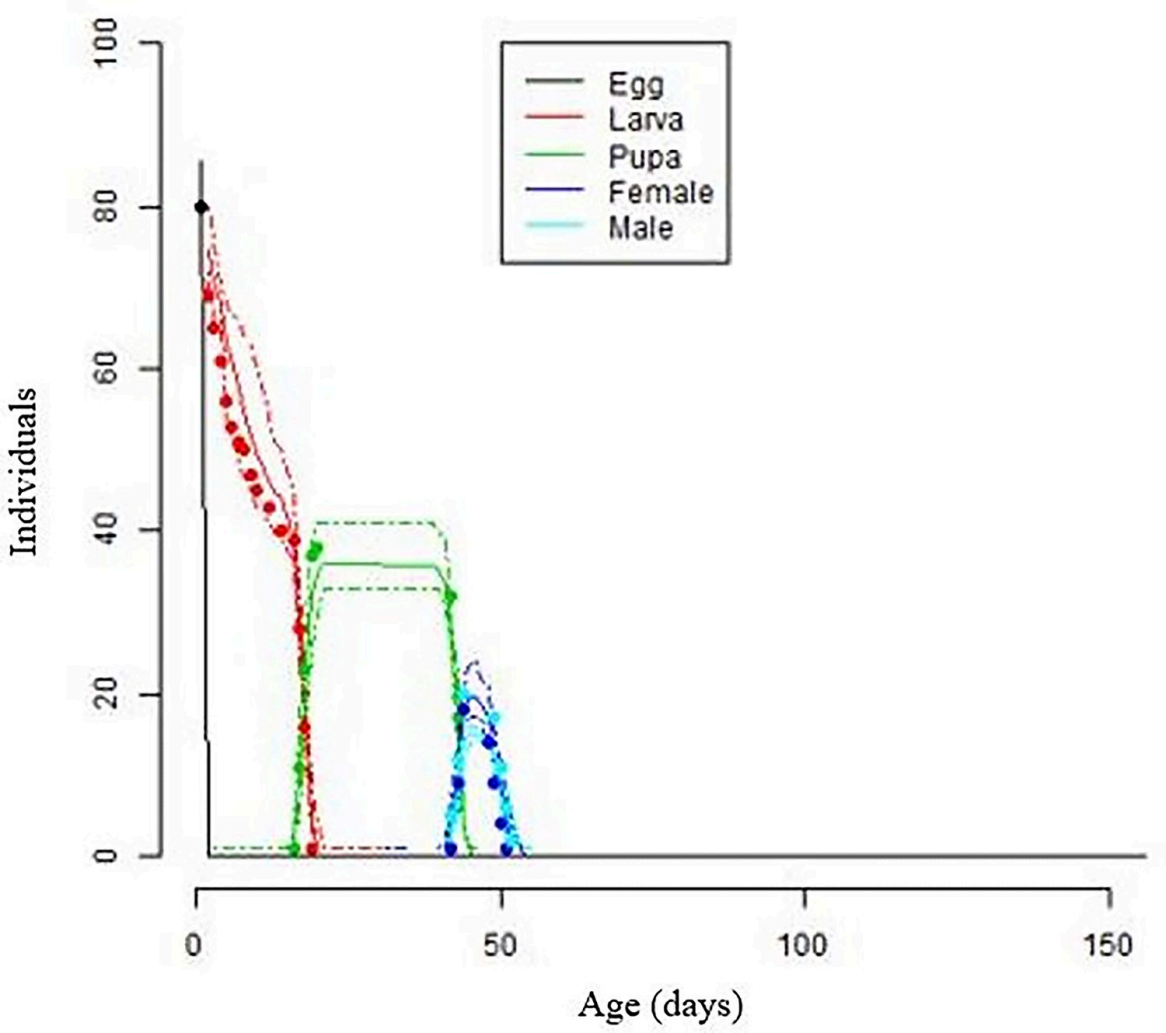
Observed and simulated *S*. *frugiperda* life stage frequencies to test temperature-dependent development models for development, survival, and reproduction. The lines reflect phenology model-simulated values for each life stage, whereas the dots represent experimental values from variable temperatures.

Temperature greatly affected *S*. *frugiperda* life table parameters. At constant 20–28°C, the intrinsic rate of increase (r_m_) was much larger than at other measured temperatures (Table 8). With 420.6and 115.6 daughters per female the gross reproductive rate (GRR) was highest and lowest at 25°C and 32°C, respectively (Table 8). At 20 and 25°C, the net reproduction rate (Ro) increased dramatically from 184.64 to 211.68, while at 30°C it declined drastically to 22.9 (Table 8). As the temperature increased, the mean generation time (Tc) dropped. FAW population doubling time (Dt), which is the time required for the pest to double, followed a different trend compared to GRR, Ro, and Tc, in that the Dt was shortest with3.28at 28°C. The finite rate of growth (λ) varied from 1.11 at 32°C to 1.23 at 28°C (Table 8). FAW developed, survived, and reproduced best between 25and 28°C, with strong population growth, short generation times, and rapid population doubling (Fig 6). These results indicate that there are statistically significant differences in the means of each parameter across different temperature groups, as evidenced by the low p-values and high F-statistics for each parameter. This significance is especially strong for the mean generation time (T_lambda), which shows an exceptionally high F-statistic, indicating a very significant difference in means across temperatures.

**Table 8:** Simulated life table parameters of *S*. *frugiperda* reared at five constant temperatures. The intrinsic rate of increase (r_m_), gross reproduction rate (GRR), net reproduction rate (Ro), mean generation time (Tc in days), doubling time (Dt) in days, and finite rate of increase (λ).

Temperature	r_m_	Ro	GRR	Tc	λ	Dt
20	0.121059	184.64	370.0843	43.10626	1.128692	5.725690
25	0.189055	211.68	420.6051	28.32548	1.208107	3.666377
28	0.210968	125.68	378.9192	22.91217	1.234873	3.285553
30	0.157310	22.90	131.2514	19.90422	1.170359	4.406245
32	0.108643	8.62	115.4717	19.82727	1.114764	6.380070
Statistics	F: 57.80 p < 0.001	F: 87.76 p < 0.001	F: 30.51 p < 0.001	F: 3536 p < 0.001	F: 57.80 p < 0.001	F: 14.98, p < 0.001

### Model validation

Fig 6 and Table 9 show immature stage development and mortality of FAW, as well as life table parameters from the experiment at varying temperatures and those predicted from the fitted phenology model. The development, mortality, and population growth characteristics matched observed and modeled values (Table 6). The simulated development times were 1.99, 16.22, and 25.06 days, compared to the observed 1.0, 16.0, and 25.18 days for egg, larva, and pupa, respectively (Table 9). Observed and simulated life table parameters were also very comparable (Table 9). The fitted models were satisfactory since the observed *S*. *frugiperda* developmental stage frequencies matched simulated values (Fig 6).

**Table 9:** Comparative analysis of area changes for fall armyworm risk indices under current, RCP 2.6, and RCP 8.5 climate scenarios for 2050 and 2070.

Year	Index	Scenario Change	Low Class Change (sqkm)	Medium Class Change (sqkm)	High Class Change (sqkm)
2070 2050 2070 2050 2070 2050 2070 2050 2070 2050 2070 2050	GI GI GI GI AI AI AI AI ERI ERI ERI ERI	Current Vs RCP 2.6 Current Vs RCP 2.6 Current Vs RCP 8.5 Current Vs RCP 8.5 Current Vs RCP 2.6 Current Vs RCP 2.6 Current Vs RCP 8.5 Current Vs RCP 8.5 Current Vs RCP 2.6 Current Vs RCP 2.6 Current Vs RCP 8.5 Current Vs RCP 8.5	-2,187,491 -516,099 -1,081,615 -1,828,710 -2,607,380 -1,258,143 -1,405,622 -230,7233 1,064,055 -766,372 326,874 665,854	1,321,666 -187,627 734,988 1,436,525 715,680 -67,913 973,613 1,118,572 -2,152,764 -426,204 -1,026,818 -1,332,984	325,578 163,479 346,627 392,185 1,351,796 122,090 432,328 1,808,958 548,463 652,329 699,944 667,129

### Mapping of the potential distribution of *S*. *frugiperda* under current climate scenarios

#### Activity index

**Fig 7:**
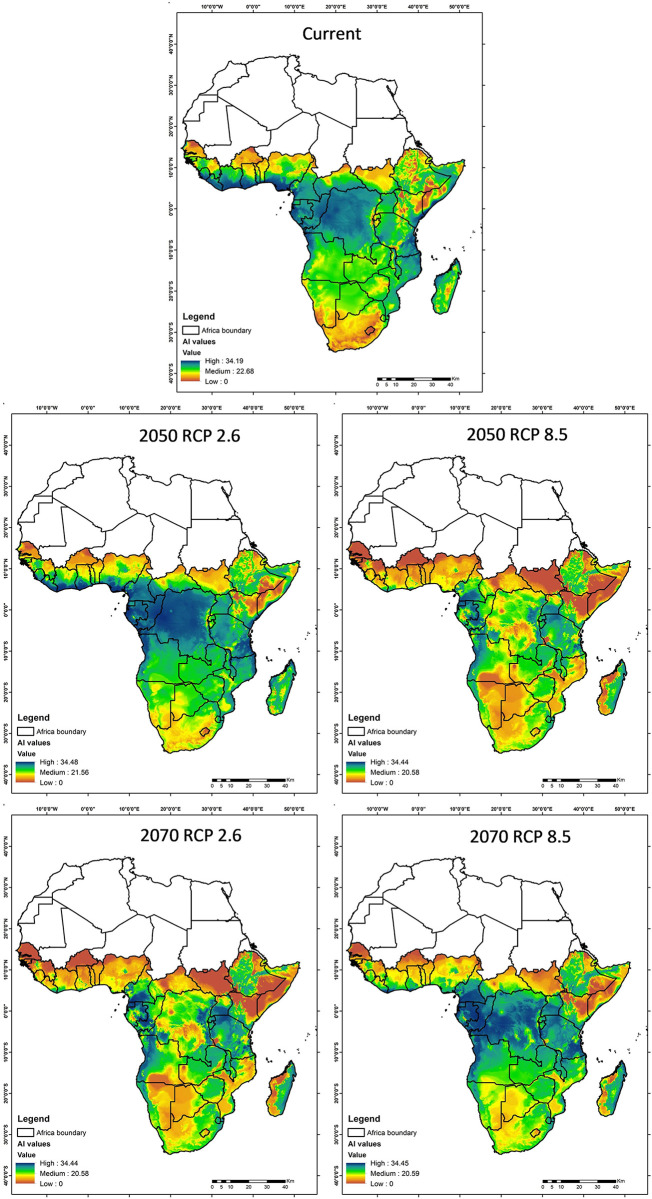
Illustration of the activity index (AI) of the fall armyworm in Africa for the current scenario, 2050 and 2070, under two contrasting climate change scenarios: RCP 2.6 (more ambitious climate mitigation) and RCP 8.5 (more pessimistic climate change). The value ranges from high (blue), medium (green) and low (orange).

The Activity Index (AI) is depicted in Fig 7, illustrating the potential activity levels of the fall armyworm on the African continent. In the current scenario, the AI reaches its peak in the equatorial regions, signifying substantial activity in areas where climatic conditions are most favourable for the pest. Projected data for 2050 under the RCP 2.6 scenario indicate a notable decline in AI, particularly in central Africa. This suggests that with aggressive climate mitigation, the activity of fall armyworms could see a significant reduction. Conversely, under the RCP 8.5 scenario for the same year, high AI levels persist, implying the possibility of ongoing or exacerbated pest activity in the absence of substantial climate action. Moving ahead to 2070, the AI projections diverge more starkly between the two scenarios: RCP 2.6 forecasts a substantial decrease in high AI areas, underscoringg the long-term benefits of climate mitigation. In contrast, RCP 8.5 anticipates the persistence of high AI levels across a broader region, emphasizing the potential for increased fall armyworm activity if current emission trends continue.

#### Establishment risk index

**Fig 8:**
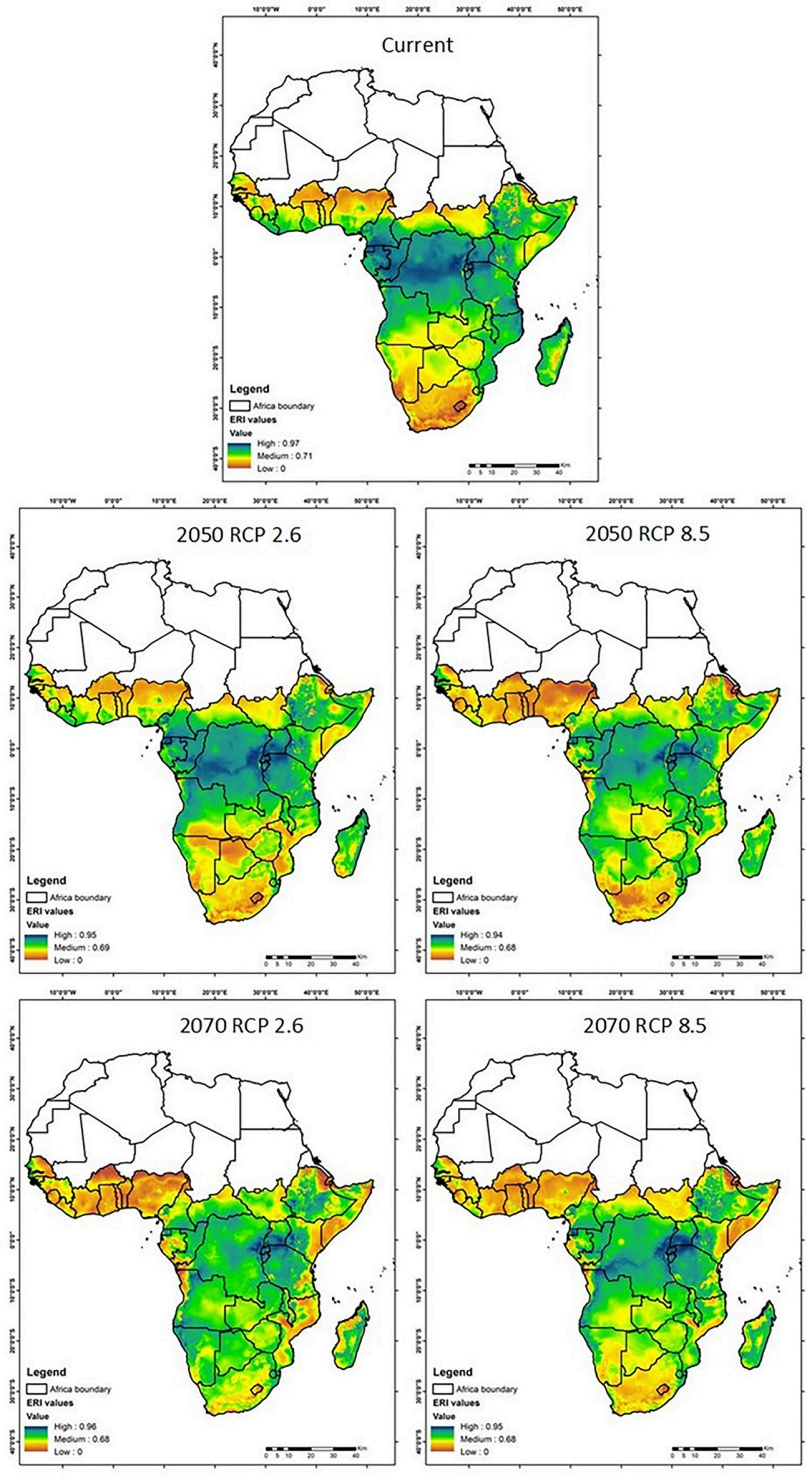
Illustration of the Establishment Risk Index (ERI) of the fall armyworm in Africa for the current scenario, 2050 and 2070, under two contrasting climate change scenarios: RCP 2.6 (more ambitious climate mitigation) and RCP 8.5 (more pessimistic climate change). The value ranges from high (blue), medium (green) and low (orange).

Fig 8 represents The Establishment Risk Index (ERI) map, offering insights into the potential areas where the fall armyworm may establish populations. Currently, the highest risk of establishment is concentrated in the equatorial belt. In the year 2050, within the optimistic RCP 2.6 scenario, there is a significant shift towards lower risk categories, particularly evident in central Africa. This shift suggests that proactive climate measures could reduce the regions susceptible to FAW permanent occurrence. However, under the RCP 8.5 scenario, the risk of permanent occurrence remains persistently high and, in fact, expands. This expansion implies an increases prevalence of suitable habitats for the pest’s establishment, driven by warmer conditions and inadequate mitigation efforts. Fast forwarding to 2070, under RCP 2.6 scenario, the trend of diminishing high-risk areas persists, underscoring the continued significance of sustained climate mitigation efforts. In contrast, the RCP 8.5 scenario shows an expansion of high-risk zones, suggesting the potential for an increased prevalence of fall armyworm occurrence with ongoing climate change.

#### Generational index

**Fig 9:**
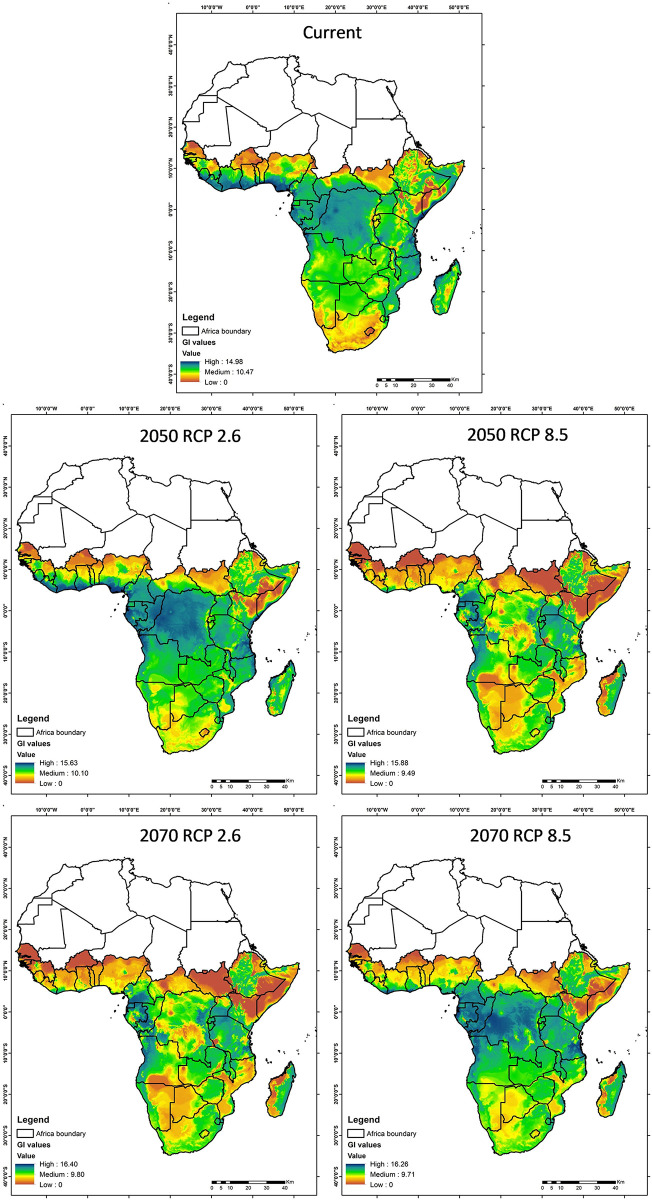
Illustration of the Generation Index (GI) of the fall armyworm in Africa for the current scenario, 2050 and 2070, under two contrasting climate change scenarios: RCP 2.6 (more ambitious climate mitigation) and RCP 8.5 (more pessimistic climate change). The value ranges from high (blue), medium (green) and low (orange).

Fig 9 portrays the Generation Index (GI), which projects the potential number of fall armyworm generations across Africa, considering both current and projected climate conditions. In the current scenario, medium to high GI values are predominantly observed in the equatorial regions, indicating a conducive environment for the pest’s reproduction. Looking ahead to the year 2050 under the RCP 2.6 scenario, there is a noticeable reduction in areas with high GI values, primarily in central Africa. This suggests that proactive climate mitigation measures could lead to reduction in the number of fall armyworm generations. However, under the RCP 8.5 scenario, the reduction in high GI areas is less pronounced, indicating that without sufficient climate action, the fall armyworm may continue to thrive. Moving to 2070, projections under RCP 2.6 suggest a continued decrease in regions with high GI, emphasizing the positive impact of long-term climate strategies. Meanwhile, RCP 8.5 indicates an increase in regions with high GI, underlining the possible repercussions of inadequate climate policies on the reproductive cycles of fall armyworms.

1. Activity index
2. Establishment risk index
3. Generation index

Fig 8. Illustration of the Establishment Risk Index (ERI) of the fall armyworm in Africa for the current scenario, 2050 and 2070, under two contrasting climate change scenarios: RCP 2.6 (more ambitious climate mitigation) and RCP 8.5 (more pessimistic climate change). The value ranges from high (blue), medium (green) and low (orange).

### The area of the indicies was represented in square kilometers unit (sqkm)

Complementing the spatial trends illustrated in Figs 7–9 and Table 9 quantifies the changes in suitable habitat areas for the Fall Armyworm (FAW) risk indices under current and projected climate scenarios. The area changes, expressed in square kilometers, reflect the potential increase or decrease in FAW risk across the African continent. Under the RCP 2.6 scenario, we observe a general decrease in High-risk areas for all indices by 2070, indicating the efficacy of climate mitigation strategies. Conversely, under RCP 8.5, the persistence or increase in High-risk areas for AI and GI by 2070 suggests that without substantial climate action, FAW could maintain or expand its presence. These quantitative changes align with the spatial patterns depicted in the figures and underscore the significant implications of climate action for FAW management.

## Discussion

The impact of global warming extends to insect pests, host plants, and their natural enemies [31,32]. A critical aspect in assessing the effects of climate change on pest populations is understanding their phenology. Several studies have delved into the phenology of *S*. *frugiperda’s* and its response to temperature variations. For instance, in He et al. [22], the authors examined the influence of temperature on various stages *S*. *frugiperda’s* including incubation, larval development, and pupation. Meanwhile, Ashok et al. [21] examined how temperature affects multiple aspects of FAW’s, including its growth, survival, reproduction, distribution, abundance, and overall population dynamics. Despite these valuable contributions, previous studies did not fully simulate the entire life cycle of *S*. *frugiperda’s* under varying temperature conditions, as demonstrated in Chen et al. [5].

The study utilized the ILCYM software to analyze how temperature affects *S*. *frugiperda*’s life cycle, population growth rate, and range extension in different African climates. Thus, we simulated FAW’s whole life history using phenology modeling and determined the thermal requirements of its different life stages, including mapping the current pest phenology in different agro-ecological zones in Africa, unlike prior studies that merely simulated the species’ development time and development rate at constant temperatures [21–23]. ILCYM, and non-linear models (Logan 5 and Hilbert & Logan 2 functions) fitted the data to estimate the development upper threshold. This study demonstrated that Fall armyworm’s (FAW) life cycle depends on temperature and that different phases have varied thermal needs. ILCYM allowed us to model the whole life history of *S*. *frugiperda* at different temperatures, unlike earlier research [22,23]. The pupal stage of the *S*. *frugiperda* life cycle requires a temperature range between 20°C and 32°C. Extreme temperatures killed immature stages, and fecundity peaked at 23–25°C. This shows the importance of temperature requirements for pest management and species dispersion projections under climatic change across Africa. Prasad et al. [33] simulated *S*. *frugiperda*’s phenology using ILCYM, but only in India.

The results for *S*. *frugiperda* egg and larval development align with the findings of Ashok et al. [21] and He et al. [22], but they differ from those reported by Du Plessis et al. [23] for larvae and pupae. Experimental procedures and maize varietal choice likely explain these disparities [22]. Logan 5 and Hilbert & Logan 2 functions [8] fit egg and larva development best, while Logan 1 fits pupal development best. Logan has two advantages. First, it considers temperatures above and below ideal. Second, it estimates the upper development threshold [34]. *S*. *frugiperda* mortality rates were highest at 32°C and lowest at 20°C because temperature affects insect physiology, biochemistry, and metabolism [9]. We used the Wang 1 and Wang 2 functions to accurately forecast *S*. *frugiperda* immature stage mortality and calculate the optimal temperature (Topt) for each life stage, where mortality was lowest. The study indicated that the egg had the lowest mortality rate at 21.64°C, the larva at 24.79°C, and the pupa at 18.09°C. Other research examined FAW’s phenological stage mortality using the Wang model. Prasad et al. [33] predicted FAW first to fifth instar mortality and reported greater rates at earlier developmental stages and higher temperatures. The Wang model observed that higher temperatures accelerated development and increased mortality, notably at the egg and pupal phases [35]. These studies found that cooler locations and higher elevations had greater FAW phenology and mortality rates. These studies show that the Wang model can forecast pest mortality rates in different phenological stages and regions, which might inform pest management techniques.

*S*. *frugiperda* fecundity was high at 23–25°C and dramatically dropped at 32°C. High temperatures reduce female lifespan and maturity, which lowers fertility [36]. Schlemmer [37] found that FAW reproduction was optimum at 22°C. Temperature affects *S*. *frugiperda* bionomics and life table parameters like intrinsic rate of natural increase (rm). rm (0.10) at 32°C inhibited *S*. *frugiperda* population growth. Xie et al. [38] discovered a mean generation time of 40.92 days for *S*. *frugiperda* larvae fed maize leaves, whereas He et al. [22] and Ashok [21] found 35.47 and 36.63 days at 25°C and 27°C, respectively. Our study reported a mean generation period of 28.32 days at 25°C, similar to Sotelo-Cardona et al. [39] and Wang et al. [28]. Wang et al. [28] showed greater net reproductive values and rm (0.2056) and (1.2283) of *S*. *frugiperda* when fed maize. Russianzi et al. [40] found a 4.49-day FAW doubling time on maize, similar to our 4.40-day finding. However, due to its shorter development time and higher net reproduction rate, *S*. *frugiperda* can rapidly increase its population and potentially more than double its numbers [41].

In our analysis of the potential expansion of suitable habitats for FAW under RCP 2.5 and 8.5 scenarios for 2050 and 2070, we observed significant contrasts in Activity Index (AI) values, reflecting the profound influence of emission trajectories on pest dynamics. Under the conservative RCP 2.6 scenario, we note a potential reduction in FAW threats, particularly in Central Africa, emphasizing the benefits of aggressive climate mitigation efforts. Conversely, the RCP 8.5 scenario warns of increased FAW prevalence, highlighting the dire consequences of insufficient climate action. These findings, resonating with the works of Ramasamy et al. [42], Susheela et al. [43], and Choudhary et al. [44], underscore the critical interplay between climate policies, pest dynamics, and food security, advocating for urgent and robust climate strategies to mitigate the burgeoning pest-related challenges in a changing climatic landscape.

Nevertheless, it’s important to note that biotic factors, such as interactions with other host plants, migratory events, natural enemy activity, biological control measures, and adaptations to new cropping systems, can have a significant impact on pest dynamics and abundance. Therefore, current prediction models come with inherent uncertainties. Previous research has indicated that temperatures above 30°C can decrease fall armyworm populations [19,45]. Higher temperatures may also boost FAW predators and parasitoid wasps, which can control their population. Chen et al. [46] found parasitoid wasp activity increases at high temperatures. However, temperature determines *S*. *frugiperda*’s spread and prevalence. The insect thrives in warm, humid circumstances, however, the temperature range in North Africa may be less favorable than in sub-Saharan Africa [19]. Yan et al. [45] found that climate change may affect fall armyworm distribution and population dynamics. As a result, the risk of FAW infestation may decrease in some locations while persisting in others.

In light of our findings, it is imperative to understand that the relationship between temperature and the population dynamics of *S*. *frugiperda* is multifaceted and not uniformly linear. While our study underscores the significant role of temperature in influencing the life history and distribution of *S*. *frugiperda*, it is crucial to recognize that higher temperatures do not necessarily guarantee an increase in their populations. Indeed, beyond certain thresholds, high temperatures may exert detrimental effects, either through direct physiological stress on the pest or indirectly by enhancing the efficacy of natural predators. This intricate interplay underscores that in some scenarios, especially at temperatures exceeding the pest’s optimal range, we might observe a reduction in population levels. Therefore, our study emphasizes the need for pest management strategies that consider the complex and sometimes counterintuitive interactions between S. frugiperda, temperature, and other environmental factors. [35,47]. Thus, successful pest management must address FAW’s intricate interactions with its environment, including temperature and natural enemies. These findings will help create effective FAW management techniques to lessen its impact on maize output and increase food security in affected African regions.

## Conclusion

The study demonstrates the crucial role of temperature in the life history of *S*. *frugiperda*. It highlights the importance of determining thermal requirements for accurate pest management and species distribution predictions under climatic change. By incorporating the concept of degree days into our analysis, we have gained a more nuanced understanding of how temperature influences the development and spread of FAW. Our findings underscore the critical importance of comprehending temperature’s impact on insect population dynamics, which, in turn, will facilitate the development of more effective pest management strategies. Detailed information on the thermal requirements of different life stages of FAW can be used to predict its distribution and occurrence under different temperature conditions across many important maize-producing regions of Africa. Our findings also underline how important it is to properly understand the impact temperature has on insect population dynamics and which as a result will facilitate the development of effective pest management strategies.

## Supporting information

S1 File (ZIP)

S2 File (ZIP)

S3 File (ZIP)

S4 File (ZIP)

S5 File (ZIP)

S6 File (ZIP)
